# The efficacy of interactive communication interventions for motivating blood donation: A systematic review

**DOI:** 10.1111/vox.70263

**Published:** 2026-04-12

**Authors:** Louisa Boult, Tom Douglass, Madeleine Tremblett, Jack B. Joyce

**Affiliations:** ^1^ Nuffield Department of Primary Care Health Sciences University of Oxford Oxford UK; ^2^ Institute for Public Health and Wellbeing University of Essex Colchester UK; ^3^ School of Social Sciences University of the West of England Bristol UK

**Keywords:** blood donation, communication interventions, systematic review

## Abstract

**Background and Objectives:**

The global blood product shortage is a persistent problem that urgently needs addressing. Research has investigated the effects of incentives, different interventions and different modes of communication on promoting blood donation; yet, there has been no systematic review comparing interactive communication–based interventions. Identifying effective methods to recruit and retain blood donors could inform future intervention design and improve resource allocation, thereby reducing strain on blood‐product supply.

**Materials and Methods:**

This review brings together relevant literature found on Medline, CENTRAL, Embase and PsychINFO to address the following questions: ‘What are the most effective interactive interventions for promoting blood donation?’ and ‘Are these different between encouraging first‐time and returning blood donors?’ A total of 36 studies were included in this review.

**Results:**

Overall, the quality of research on interactive communication–based interventions was poor, with several studies lacking sufficient detail to make clear conclusions. Evidence for three common interventions (phone calls, educational sessions and motivational interviews) was conflicting. Additionally, there was limited research into web‐based interventions and recruitment of first‐time donors.

**Conclusion:**

Reporting of communication interventions for blood donation could be improved, and our review suggests that a more heterogeneous approach to donor recruitment may be advantageous.

**Highlights**

- This is a systematic review of communication interventions motivating blood donation.
- A heterogenous approach to donor recruitment appears advantageous.
- Communication interventions can be low‐cost, quick, acceptable and effective.

## INTRODUCTION

Globally, 118.5 million units of blood are donated per year, yet this supply is insufficient to meet international demand [1]. As there is no synthetic blood substitute, health services rely entirely on people donating [2]. To illustrate the scale of this need, in the United Kingdom alone, 4300 blood donations are required every single day to meet hospital needs [3]. The World Health Organization deems regular, voluntary, non‐remunerated blood donations to be safest, as these donors have the lowest prevalence of blood‐borne infection [4]. Yet, in 54 countries, over 50% of blood comes from family, replacement or paid donors [1]. Additionally, whole blood donations can be given only every 56 days [5], and donations have a shelf life of 35 days [6]. Consequently, strategies are needed both to recruit new donors and support sustained repeated donation in order to maintain a safe, sufficient and resilient blood supply.

Several reviews have been dedicated to increasing blood donation [7], often investigating the use of incentives [8] and interventions [7] for promoting donation. Incentives—concessions or payments to reward blood donation—can be helpful for initiating blood donation but they lose efficacy as motivation changes throughout a donor's career [9, 10]. Some reviews have examined communication strategies to increase blood donation across different contexts [7, 11, 12] and have found motivational, theory‐based communication interventions and reminders as potentially effective for increasing donation. While these reviews generally support the efficacy of communication interventions, they amalgamate different modalities and do not isolate the specific communication components, the ‘active ingredients’, responsible for the observed effects. Moreover, recent systematic reviews of motivational interviewing, a prototypical theory‐based communication intervention, suggest that its additional benefit relative to comparator communication interventions may be modest or highly context‐dependent for some health behaviours [13, 14, 15]. There is general uncertainty over whether the effectiveness of these interactive interventions is driven by the message content, theory‐specific techniques or the mechanism of engagement. To address this conceptual gap in how communication interventions have been synthesized, the current review focuses solely on interactive communication–based interventions. We define interactive communication–based interventions as those in which the core mechanism of the intervention relies on a two‐way exchange between at least two parties (e.g., face‐to‐face conversation, telephone calls, bidirectional messaging, educational awareness sessions and AI‐powered chatbots). Unlike passive mass media or broadcast social media campaigns, interactive interventions afford reciprocity, immediate feedback, and the addressing of specific donor concerns or barriers in (near) real time and may therefore operate through distinct behavioural mechanisms [16].

Evidence from other health behaviours suggests that interactive communication interventions are welcomed by patients [17], are cost effective [18] and quick [19] across several contexts. For example, for smoking cessation, very brief advice (e.g., discussing the benefits of quitting, teaching how to manage cravings) can be effective for increase uptake of behavioural support programmes [20, 21], and in weight‐loss settings, research has shown that brief opportunistic communication‐based interventions can motivate greater weight loss action [22]. Blood donation involves a series of decisions that are shaped by an individual's beliefs and emotions, as well as by social norms and practical barriers [23], and may therefore be amendable to interactive strategies that address potential concerns in real time. Emerging evidence in blood donation settings reports that similar interactive communication interventions can be effective, low cost and may improve outcomes such as intention to donate, appointment attendance and repeat donations [24]. However, to date, the specific interactive strategies used and their differential impact on donor recruitment and retention have not yet been systematically synthesized.

The primary aim of this systematic review is to map the landscape of interactive communication interventions for blood donation and determine what the most effective communicative, interactive interventions are, and whether these are different in the context of recruiting new or returning donors. Specifically, we seek to identify the types and components of interactive communication**–**based interventions that have been evaluated, and examine their effects on key outcomes, including intention to donate, first donation and repeat donation. In clarifying which interactive strategies appear most promising, this review aims to support blood services in developing and implementing scalable, evidence‐informed approaches to enhance rates of donation.

## MATERIALS AND METHODS

### Search methods

A list of search words describing interactive communication–based interventions and outcome measures relevant to promoting and retaining blood donors (see Appendix S1) was developed. For the purpose of this review ‘interactive interventions’ were defined as an exchange between at least two (not necessarily human) parties, such as a telephone, face‐to‐face or text message conversation (adapted from Albury et al. [17]). These constitute a sub‐group of communication‐based interventions. Eligible outcome measures included intention to donate blood, completed blood donation and frequency of return (Table S1). This applies to first‐time and returning donors, to allow investigation of blood donation promotion and retention. Before the search, the study was registered on the Open Science Framework [25]. An initial search conducted in August 2023 identified 1899 papers. The search was updated on 17 December 2025, yielding an additional 299 papers across databases commonly used to index communication‐based and behavioural intervention studies: Medline (1946–present), CENTRAL (Issue 2 of 12, 2024), Embase (1974–present) and PsychINFO (1806–present).

### Inclusion and exclusion criteria

We included any primary research studies regardless of methodological approach that report on any interactive intervention (per the definition above). To be included, participants had to be aged 16 years or older, feature an interactive component between at least two parties and have reported at least one outcome of (1) intention to donate blood, (2) attempted blood donation, (3) actual blood donation, (4) blood donation motivation, (5) frequency of retention and (6) change in behaviour (see Table S1 for inclusion criteria). Initially search terms included ‘plasma’ and ‘platelets’, but these were excluded after the search, since plasma and platelet donations are typically more complex, and donors have different motivations than whole blood donors [5, 26, 27, 28]. Whole blood donors valued the minimal effort and potential need of their loved ones for blood products, whereas plasma donors were motivated by a sense of pride [27].

### Screening

Papers were screened and data were extracted using Covidence [29]. Duplicates were removed automatically by Covidence and allowed for title and abstract screening and subsequent full‐text screening. Title and abstract screening was conducted independently by two reviewers (L.B., J.B.J.) using the predefined eligibility criteria. Uncertainties were discussed with the wider research team. This process yielded 207 full‐text articles, which were independently screened by pairs of reviewers (L.B., C.J. [C.J. declined the invitation to be an author on this paper] and J.B.J.), with uncertainties or conflicts resolved through discussions between all reviewers.

### Data extraction

Data were extracted (such as information on populations, interventions, comparisons, outcome measures and main findings) following data‐extraction guidance from the Cochrane Handbook [30]. After screening and data extraction, the references of each paper were used to determine whether the initial search omitted any relevant literature (snowballing).

### Quality appraisal

The quality of these papers was assessed using the mixed‐methods appraisal tool (MMAT), 2018 version, which considers the quality of five different categories of study design [31].

### Narrative review

For analysis, a summary of each included study was tabulated (see Table S5). Studies were grouped by intervention type, target population and outcome. Studies with multiple interventions were grouped separately. This ensured that the size and direction of effects were clear for comparison across groups. As we analysed the results, we critically evaluated the studies to identify important factors and methodological limitations. The quality of studies included was then evaluated to see how this affected the conclusions of the current synthesis.

## RESULTS

### Intervention characteristics

The outcome of the search is shown in Table S2. From the original search, 23 papers were included. After snowballing, an additional 13 papers were discovered, totalling 36 studies to be included. Of these, 20 interventions were randomized controlled trials (RCTs), whereas the others were quantitative non‐randomized studies (e.g., cohort studies) (*n* = 15) or qualitative (*n* = 1). The studies were conducted in several countries: 15 in the United States, 4 in India, 3 each in Canada and China, 2 in Australia and 1 each in Brazil, France, Germany, Iran, Nigeria, Serbia, Sierra Leone, Switzerland and Turkey. Various methods of communication were used; the most common were telephone calls or in‐person interventions. Publication years ranged from 1977 to 2024.

Interventions were categorized according to the primary modality of communication as described by the original study authors. Categories included telephone‐based, in‐person conversational, educational or interactive awareness sessions, multi‐component (e.g., a call, education session and/or education session) and web‐based. Telephone‐based and conversational interventions typically involved scripted or semi‐structured discussions; education sessions focused on the provision of information content, often delivered in a group‐based setting; web‐based interventions included live chats via a website; and multi‐component interventions combined more than one delivery modality. These categories are not mutually exclusive and reflect how interventions are operationalized in the literature. Most studies targeted previous donors for blood donor retention (*n* = 17) or investigated both initiating donation and retention (*n* = 7). Only two studies specifically aimed to recruit new donors. Other studies did not specify who they were targeting (*n* = 10).

### Participant characteristics

The mean age of participants in the studies that reported age (*n* = 16) was 29.6 years. Studies were commonly conducted with university/college students and high (secondary) school students (*n* = 10). Participant's gender was reported in 18 studies; most participants were female.

### Risk of bias assessment

The quality of each study was assessed using the MMAT [31] and scored each criterion as ‘yes’, ‘no’ or ‘inconclusive’ (Table S3). Generally, the quality of the studies was poor, although most studies had complete outcome data (at least 50% response rate). At least one criterion was unclear or unmet in 35/36 studies. Of these, 18 had three or more unclear or unmet criteria.

For the non‐randomization studies (*n* = 15) two key weaknesses were identified: 11/15 lacked consideration of confounding variables, and 7/15 did not consider how representative the participants were of the target population.

Of the 20 RCTs, it was unclear for 14 whether they had appropriate randomization. Additionally, for 16, it was either confirmed that groups were not comparable at baseline, or it was unclear. Given the nature of the intervention, it is difficult for the assessors to remain blind.

### Funding and declarations of interest

The majority of studies reported no funding. Those that did (*n* = 17) were supported through charitable organizations or university, departmental or governmental grants. No study reported a competing interest. This information is detailed for each study in Table S5.

### First‐time recruitment

Only two interventions solely sought to recruit new donors [32, 33]. One found that addressing knowledge gaps, potential concerns and misconceptions through interactive awareness sessions positively increased willingness to donate blood [32]. This included a questionnaire to identify misconceptions, emotional barriers or concerns about blood donation followed by a 2‐h session in which those issues could be addressed. The other intervention featured a call plus an email brochure, which saw a significant increase in appointment attendance [33].

### Retaining donors

Sixteen out of 36 studies solely investigated methods of retaining previous blood donors [34, 35, 36, 37, 38, 39, 40, 41, 42, 43, 44, 45, 46, 47, 48, 49]. See Table S4 for results summary.

#### Outcomes of phone calls

Most telephone studies used donation attempt as an outcome. Phone calls with motivational interviews as well as structured conversations designed to explore and resolve ambivalence by addressing beliefs, motivations and barriers significantly increased motivation for re‐donation, as well as the rate of return within 60 weeks [38]. Another study found that phone calls made a few days before donors were next eligible to donate had a significant positive effect on attempts to donate in a 12‐month follow‐up period [42]. Ou‐Yang et al. measured donation activity for 1 year; average‐treatment‐effect‐on‐treated analysis found that telephone calls significantly improved the re‐donation rate. This study included both whole blood and platelet donors; however, across all groups, almost all cases of donors donating again were from those who donated whole blood [45]. Their similar earlier study found telephone, text messaging and control groups had significantly different re‐donation rates over a 7‐month follow‐up period, especially between the phone group and the control group. This study also investigated reactivating whole blood and platelet donors, but only 1 of the 37 who re‐donated was a platelet donor [46]. Porto‐Ferreira et al. investigated the effects of receiving a text, letter or phone call (all containing similar information) on 30‐day return rate, specifically in donors who had previously been deferred for serologically reactive screening tests [47] (this is for donors found positive for a transfusion‐transmissible infection, such as human immunodeficiency virus, hepatitis B, hepatitis C, human T‐lymphotropic virus, syphilis or Chagas disease). The phone call had better odds of return than text, but the call was not better than letter. Clee and Henion found no significant difference in verbal agreements to donate or attempts to donate between four conditions (see Table S5) [36].

Three studies reported a significant increase in actual donation rate following a phone call [35, 48]. One study found that the donation rate increased 7.8% following a phone call [35], and another found the donation rate was 10.8% higher for first‐time donors after receiving a phone call [50]. Yet another study found that a phone call was significantly more effective than an email—encouraging a 14.4% increase in second donation within 6 months [48]. A study measuring donations at a blood bank facility found recruitment via a phone call was responsible for 63% recorded donations, yet only 10% of those who said they would donate did [34].

Intention to donate was another outcome measured. France et al. found that phone calls with a motivational interview were associated with significantly larger increases in intention to donate [39]. There was no significant difference between the interview and control groups with respect to donation type (whole blood or double red blood cell). Germain and Godin found that phone plus email had the highest percentage of donors register to give blood, compared to only phone or only email, which did not significantly differ [41]. This difference was only seen in men. Sinclair et al. found that adapted motivational interview was associated with greater intention to donate as well as a higher rate of attempted donation at 12 months but not at 3 and 6 months [49].

#### Outcomes of education interventions

Educational sessions had a significant impact on the rate of application to become donors. Eser et al. found that the rate of admission for blood donation was higher for university students compared to city residents following a brief group education session discussing blood donation need [37]. Students had a significantly higher rate of application to become donors after the intervention.

#### Outcomes of conversation interventions

Conversation interventions were associated with an increase in odds of donors returning. Guided conversations gave the donors the opportunity to express concerns and receive tailored encouragement. Gemelli et al. measured attempts to donate blood up to 3 months after donors had previously been deferred [40]. Novice and established donors receiving an in‐centre brochure, guided conversation plus email had increased odds of returning at 3 months, compared to controls.

#### Outcomes of web‐based interventions

Hu et al. found that participants exposed to a web‐based intervention had a significantly higher re‐donation rate compared to control [44].

#### Outcomes of multiple interventions

Hashemi et al. measured attempts at second donation within 6 months of first donation following various interventions [43]. Phone calls made a significant impact on retention only in male donors.

### Recruiting and retaining donors

A total of seven studies aimed to recruit new donors and retain previous donors [51, 52, 53, 54, 55, 56, 57].

#### Outcomes of interactive awareness sessions

Only four studies explored the use of awareness‐raising sessions on recruiting both new and returning blood donors [51, 52, 56, 57]. Bachhotiya et al. investigated this in medical students, who were significantly more willing to donate post the intervention [51]. Similarly, Chauhan et al. found an interactive awareness session for medical students was associated with increased self‐reported willingness to donate [52].

Sarason et al. investigated the effects of various presentations given to high school students—a videotape presentation increased the relative risk for donation [56]. The 1991 study compared the content of presentations delivered [57]. Presentations that included psychological content (e.g., addressing emotional barriers, personal values and beliefs) increased the actual donation rate more than the educational presentations or control [57].

#### Outcomes of phone calls

Phone calls influenced donation behaviour across all donor types, regardless of donation history. Hayes et al. investigated two strategies for encouraging blood donation: foot‐in‐the‐door, which involves a small initial request to increase compliance, with a larger one, and door‐in‐the‐face, which begins with a large request likely to be refused, making the actual request seem more reasonable [53]. Compared to control, door‐in‐face led to fewer donations, whereas foot‐in‐door increased donations among active and inactive donors as well as non‐donors.

#### Outcomes of in‐person interviews

Similarly, in‐person interviews influenced undergraduates' attitudes towards blood donation. Livitz et al. compared motivation and knowledge‐based interviews [54]. More students in the motivational interview shifted to internal motivation. That is, students reported donating because it aligned with their values or felt meaningful rather than being driven by external rewards, such as gifts or approval, which showed greater increases in intention to donate than those in the knowledge group.

#### Outcomes of web‐based interventions

Receiving tailored, computerized feedback increased the likelihood of considering blood donation. Robbins et al. investigated the use of a programme website for improving attitude towards donation in Black adults, with personalized computer‐tailored feedback being delivered via a website [55]. Participants were significantly more likely to consider donation after the intervention.

### Unspecified populations

Finally, 10 studies did not describe if they were trying to recruit new or existing donors [58, 59, 60, 61, 62, 63, 64, 65, 66, 67].

#### Outcomes of multiple interventions

Three studies looked at the effects of multiple interventions at once with varying success. Charbonneau and Daigneault found that various awareness‐raising activities were able to increase the number of Black community donors from 170 in 2009 to 1582 in 2012 [58].

Jason et al. found no significant difference between the use of various media for promoting blood donation in undergraduate students [61]. For another blood drive, seven students were asked to approach five friends and five strangers and commit them to donating. Less than half of the friends and strangers agreed to donate, and even fewer donated.

Srzentić et al. encouraged university students to donate using various media, including discussions and media conversations [65]. This was associated with an increase in the average number of students at blood drives, as well as the number of students donating blood.

#### Outcomes of phone calls

Phone calls appeared successful at improving outcomes in unspecified populations. Ferrari et al. found that 93% of subjects phoned attended a blood drive, compared to 56% of those not called [59]. However, the subjects phoned had already pledged to donate—the phone call was asking if they were still intending to go and thanking them.

LaTour and Manrai found that a letter and phone call together had a significantly positive effect, but only a marginally significant difference between conditions with and without the normative influence (i.e., social pressure or cues about what others expect) when no additional information was provided [62].

Lipsitz et al. found an experimental call significantly increased attendance of college students at a blood drive [63]. In another experiment, they found that introducing external social pressure by prompting participants to consider how others would view their donation behaviour significantly increased donation attempts compared to control.

#### Outcomes of education

Education may have contributed to increased annual donations, as well as improved attitudes towards blood donation. Sengeh et al. measured an increase in annual donations following several interventions at a hospital, including an educational campaign in the community [64].

Ugwu et al. recorded the effect of an educational workshop on medical students' attitude to blood donation; they were more willing to donate blood and encourage their friends/family to donate post the intervention [67].

#### Outcomes of other interventions

Informal interviews, described as semi‐structured discussions, may have contributed to blood donations, although the relationship is unclear. Grassineau et al. measured the effect of informal interviews on blood donation in a Comorian immigrant community [60]. During the study, 92 people volunteered to donate, although it is hard to know if this was because of the intervention due to a lack of reporting detail.

Combining personal contact and mailing positively impacted intention to donate. In the Swinyard and Ray study, volunteers approached female household residents to deliver a persuasive appeal to donate, followed by increasing levels of promotional mailings [66]. Face‐to‐face contact allowed for concerns and values to be discussed in person, and intention increased more with contact plus increasing levels of mailings.

## DISCUSSION

In this analysis of 36 studies, we reviewed literature on interactive communication–based interventions for increasing blood donation among new and returning donors. Insufficient and poor‐quality evidence for recruiting solely new donors and web‐based interventions make it difficult to draw reliable conclusions. Despite the low‐level evidence, this review found addressing donors' concerns, beliefs and emotions to be beneficial. Previous donation history, age and male sex were associated with positive outcomes. We found inconsistent evidence for several interventions, including educational awareness sessions, phone calls and motivational interviews. Our findings align with previous systematic reviews reporting heterogeneous effects of communication interventions and highlighting methodological limitations in the evidence base [7, 12, 23]. The current review suggests that the modality of the reminder could be significant and that the added value of interaction could depend on the script of the conversation. Interpretation of comparative effectiveness should, however, be approached cautiously, given the substantial heterogeneity in intervention content, comparators and outcome reporting across the included studies.

### Implications for the future

#### More evidence needed

One possible reason why results for phone call efficacy were mixed is a difference in comparators; three of the studies that found phone calls to be insignificant compared them against other interventions with digital/remote modes of message delivery, like text messages and emails [41, 47, 61], whereas all studies that found phone calls to be significant used no‐intervention controls [45, 46]. One trial comparing a phone call intervention to a non‐intervention control group found a higher rate of return donation [50]. The implication of this is that, to have a stronger evidence base, future studies could have a non‐intervention control group to allow for more meaningful comparison of results. This also points to an important difficulty—it is hard to untangle the effect of the means of receiving an intervention from the effect of simply receiving a message in the first place. General reminders have been shown previously to be helpful in the context of sleep interventions [68] and are cited in blood donation reviews as a retention tool [69, 70]. However, unlike broader reviews, which have grouped reminder types together, our analysis indicates that interactive reminders could be more advantageous. Some studies in the present review found phone calls to have a significant effect only as a follow‐up to letters or emails [33, 41, 62]. Upon critical assessment, it is possible that the additional reminder, not the phone call per se, improved outcomes in these groups.

A second reason for this inconclusiveness could be a difference in follow‐up period. Porto‐Ferreira et al.'s study had a short follow‐up period so a different trend could emerge, given sufficient time [47]. In Sinclair et al.'s (motivational interview) study, the intervention started having a significant effect only at 9 months and becoming more obvious at 12 months post intervention [49]. Interactive communication–based intervention studies could allow for more adequate follow‐up periods to observe longer term changes in donation behaviour.

A third important factor responsible for the mixed results is the difference in intervention length. This was particularly prominent in motivational interviews; a 1‐h‐long meeting was ineffective at retaining donors [43], yet two, shorter interviews were successful [49, 50, 54]. The current review seems to suggest that longer forms of motivational interview are no more effective than quick communicative interventions; there is no need for practitioners to learn a lengthy, complex tool to recruit donors. Future research could build upon the evidence for shorter interventions being equally if not more beneficial than longer ones, as seen in the context of weight loss [19, 22]. While motivational interviews are seemingly successful for blood donation and some other contexts [38, 39, 49, 54, 71], the wider literature is inconsistent and suggests that motivational interviews have no or unclear effects in other contexts [13, 14, 71]. As a common interactive communication–based intervention, further empirical work could explore how and why motivational interviews appear effective for blood donation compared to other contexts.

#### Better reporting of content

Intervention content affected motivational interviews. Successful interviews used trained personnel to deliver open‐ended questions, affirmations and reflections to address individuals' motivation for donating and any barriers or concerns [49, 54]. These were face‐to‐face interviews; in‐person teaching is acceptable [72] and the benefits are known [72, 73, 74]. Yet, it was difficult to assess the importance of the content in phone calls because failure to clearly describe methods was common. Only six studies provided a script [45, 47, 48, 53, 59, 66] and eight mentioned there was a script but only provided a vague description [33, 38, 39, 49, 50, 54, 56, 57, 62]. It is generally unclear how well researchers adhered to these scripts. Despite this, a common successful component of phone calls is taking an interest in donors' individual perspectives and emotions [38, 39, 48, 49, 50]. This is consistent with previous communication‐related reviews, which suggest that interventions explicitly addressing concerns, beliefs and responses were more likely to report better outcomes than purely education or informational interventions [7, 23]. Indeed, Bagot et al.'s [69] review on the retention of first‐time donors concludes that individualized experiences may be of benefit. Work on the language and delivery of blood‐related communication interventions is absent, yet it appears these factors strongly affect intervention efficacy. Communication interventions could incorporate conversation strategies expressing interest in the recipients' beliefs, concerns and feelings. To allow direct comparison and identification of which components work best, future studies could be clearer about the content of the intervention and/or undertake empirical analysis of how the communication is delivered in practice.

##### A heterogeneous approach to blood donor recruitment

The current review found a positive association between educational/awareness sessions and donation behaviour [32, 51, 52, 67]. However, all these studies were conducted on students, and the association was not seen for city residents [37]. It might therefore be concluded that these results were primarily a consequence of the demographics; students elect to continue learning and embrace new ideas. Medical students especially dedicate time, money and effort for the purpose of helping others and may feel a social responsibility to donate blood [75]. Some evidence suggests that active learning is successful in students [76] and that educating the public increases the likelihood of donation [77]. Consequently, studies targeted at people of all educational levels may benefit from including an educational component.

One might think older people would donate less blood, since previous research suggests that our ability to imagine what other people are thinking, including thoughts different from our own, declines with age [78]. This review found age and donor experience were generally positively associated with successful outcomes. This fits with previous blood donation literature and systematic reviews of donor determinants, suggesting older donors are more likely to re‐donate and that donation history is positively associated with future return [79, 80, 81]. Interactive communication–based interventions tailored to specific demographics could therefore be advantageous given the strong influence of demographic predictors as identified in other reviews [70, 82]. The ageing blood donor population and decline in the number of first‐time donors illustrate the importance of recruitment heterogeneity [83], targeting younger people to replace donors as they retire.

This need for diverse approaches is exemplified by considering the sex of donors. Studies have found women to be less likely to donate [84]. Blood donation is contraindicated during pregnancy and lactation [85]. Women are not able to donate blood as frequently as men, are more likely to be deferred due to low haemoglobin and have an increased risk of adverse effects (e.g., fainting) due to lower average weight [84]. Multiple studies in the current review highlighted that men generally respond more positively to interventions [32, 41, 44, 45, 50, 79] and respond better to interventions such as emotional letters and phone calls [43]. Interventions accounting for heterogeneity in sex may be more effective, and further research—especially into the reasons women may not donate—is warranted.

A rudimentary method to account for heterogeneity was developed by Bruhin et al., who split donors into two types [35]. Type 1 had a high baseline donation rate, with a phone call immediately increasing the probability of donation, but without habit formation. Type 2 had a lower baseline donation rate; phone calls also increased the donation probability, but they were habit‐formers. If these, or more precise, categories of donors could be identified before future interventions started—perhaps using demographic characteristics and predictive tools—this would allow tailored and more efficacious interventions to be targeted to previous blood donors, increasing their likelihood of return. Tailored interventions have already been successful in the context of smoking cessation [86]. Given the emerging role of artificial intelligence (AI) in other healthcare settings [87, 88, 89, 90], using machine learning to predict the ‘type’ of donor using their demographics and/or behaviour to prescribe the best intervention could be an advantageous avenue of research.

### Common weaknesses of studies

This review has highlighted several common weaknesses of studies recruiting blood donors using communicative interventions. Only nine studies included calculations justifying their sample size as statistically powerful (internal validity) [40, 41, 44, 45, 46, 47, 48, 50, 67]. Another problem with several populations used was generalizability (external validity). Particularly for studies using (medical) students from a single university, who are likely to be better educated than the general population, the outcomes of interventions cannot be extrapolated [32, 51, 52, 54, 56, 57, 59, 65, 66, 67].

Studies measuring intention or willingness to donate have limited use, as it is unknown whether the participants donated, or tried to but could not (e.g., due to deferral) [32, 36, 38, 39, 49, 51, 52, 54, 66, 67]. To combat the strain on blood products, effective interventions must guarantee increased actual donation rates. For studies with incentives for completing the intervention [38, 39, 50], it is possible because of social desirability bias [90], participants reported positive outlooks post intervention as they believed it was the desired response or would make them more likely to receive the money. The persistence of these limitations and the limited evidence base across reviews [7, 12, 23, 69] suggest that minimal progress has been made in researching a significant public health challenge.

## STRENGTHS AND LIMITATIONS

The current study has several strengths. Searching multiple databases increased the likelihood of discovering all available literature. The specific inclusion and exclusion criteria ensured the search remained focused on the research questions. We implemented current best practice, following the Cochrane Handbook to ensure that this review was conducted to the highest standard [91].

Nevertheless, there are limitations to the review. Many of the studies included were conducted poorly, with several weaknesses occurring repeatedly. This limits the reliability of their results in this review. Only including papers published in English may have excluded many relevant studies. This was done due to constraints of time and resources. We deliberately excluded mass‐media interventions (e.g., television, radio, newspapers) and one‐way broadcast uses of social media, as these operate through different behavioural mechanisms than interactive exchanges. This decision narrowed the scope of the review but enhanced the relevance and interpretability of the findings for the development of an interactive behavioural intervention. Future research could assess the comparative effectiveness of mass‐media and social media. Additionally, the MMAT advises using two reviewers for quality appraisal of studies [31]. There was a sole reviewer for this. We did not conduct a meta‐analysis. Also, observational studies are sometimes more likely to find an effect [92].

## CONCLUSION

The current review explored the literature on interactive communication–based interventions for recruiting and retaining blood donors. Given the desperate need for recruiting reliable blood donors and the known cost‐effective [18], quick [19], acceptable [93] and successful [17, 20, 21] nature of communicative interventions in healthcare settings, the lack of research over the last five decades into interactive interventions promoting blood donation is surprising. It is clear from this review that more evidence exploring these interventions and better reporting of study details is needed to assess the efficacy of different interventions. Finally, a shift towards more heterogeneous methods of donor recruitment could be needed to maximize donations from different populations.

## CONFLICT OF INTEREST STATEMENT

The authors declare no conflicts of interest.

## Supporting information


**Appendix S1:** Supporting information.

**Table S1.** Inclusion criteria for the search.
**Table S2**. PRISMA search flowchart produced by Covidence, showing why papers were excluded in the screening process.
**Table S3**. Risk of bias assessment. Quality of studies assessed using the mixed‐methods appraisal tool.
**Table S4**. Retaining donors—Summary of results.
**Table S5**. Summary of included studies, detailing their setting, populations, key methods, results, funding and declarations of interest.

## Data Availability

Data sharing not applicable to this article as no datasets were generated or analysed during the current study.
