# Supplementary material for: The efficacy of interactive communication interventions for motivating blood donation: A systematic review
Source: Vox Sang. 2026 Apr 12;121(8):1110–20. doi: 10.1111/vox.70263 (PMC13453930; doi:10.1111/vox.70263)
Supplement: Supplementary file 1 [file VOX-121-1110-s001.docx]

Supplementary tables

| **Population** | People aged 16 years or older. Despite the World Health Organisation suggesting blood donation should occur between the ages of 18–65, I decided to bring the age down to 16 to avoid excluding countries with lower donation ages. |
| --- | --- |
| **Intervention** | Interactive interventions between at least 2 parties. |
| **Comparison** | No restrictions. |
| **Outcomes** | Reported intention to donate blood; attempted blood donation; actual blood donation; reported blood donation motivation; frequency of retention; change in behaviour. |
| **Study type** | Any study type excluding systematic reviews, literature reviews, comments, or opinion papers.  Papers from any country (to avoid bias towards only high/middle/low-income countries) and date (to include all research as modes of communication have changed with time) were included.  Only peer-reviewed papers and studies in English are included. |

Table S1. Inclusion criteria for the search

Table S2. PRISMA Search flowchart produced by Covidence. Shows why papers were excluded in the screening process.

| **Study ID** | **Study design** | **MMAT methodological quality criterion** | | | | | | |
| --- | --- | --- | --- | --- | --- | --- | --- | --- |
|  |  | **S1** | **S2** | **1** | **2** | **3** | **4** | **5** |
| Agrawal and Tiwari, 2014[1] | Quantitative non-randomised | y | y | y | y | y | n | y |
| Bachhotiya *et al*., 2014[2] | Quantitative non-randomised | y | y | y | y | y | x | y |
| Bharatwaj *et al*., 2012[3] | Quantitative non-randomised | y | y | y | y | y | y | y |
| Bruhin *et al*., 2015[4] | Quantitative randomised controlled trial | y | y | y | x | n | x | y |
| Charbonneau *et al*., 2015[5] | Qualitative | y | y | y | x | x | n | n |
| Chauhan *et al*., 2018[6] | Quantitative non-randomised | y | y | y | y | y | n | y |
| Clee and Henion, 1979[7] | Quantitative non-randomised | y | y | x | y | y | n | y |
| Eser *et al*., 2010[8] | Quantitative non-randomised | y | y | n | y | y | n | y |
| Ferrari *et al*., 1985[9] | Quantitative randomised controlled trial | y | y | x | y | y | n | y |
| France *et al*., 2017[10] | Quantitative randomised controlled trial | y | y | x | x | y | x | y |
| France *et al*., 2016[11] | Quantitative randomised controlled trial | y | y | x | x | y | x | y |
| Gemelli *et al*., 2022[12] | Quantitative randomised controlled trial | y | y | x | n | y | x | y |
| Germain and Godin, 2016[13] | Quantitative randomised controlled trial | y | y | x | y | y | x | y |
| Godin *et al*., 2011[14] | Quantitative non-randomised | y | y | y | y | y | y | y |
| Grassineau *et al*., 2007[15] | Quantitative non-randomised | y | y | y | y | n | n | n |
| Hashemi *et al*., 2017[16] | Quantitative randomised controlled trial | y | y | y | x | y | x | y |
| Hayes *et al*., 1984[17] | Quantitative randomised controlled trial | y | y | x | x | n | x | y |
| Hu *et al*., 2022[18] | Quantitative randomised controlled trial | y | y | x | n | y | n | y |
| Jason *et al*., 1984[19] | Quantitative randomised controlled trial | y | y | x | x | x | n | n |
| LaTour and Manrai, 1989[20] | Quantitative non-randomised | y | y | x | y | y | y | y |
| Lipsitz *et al*., 1989[21] | Quantitative randomised controlled trial | y | y | y | x | y | y | y |
| Livitz *et al*., 2017[22] | Quantitative non-randomised | y | y | n | y | y | n | y |
| Marsano *et al.,* 2024[23] | Quantitative randomised controlled trial | y | y | y | y | y | n | y |
| Masser *et al*., 2016[24] | Quantitative randomised controlled trial | y | y | x | x | y | x | y |
| Ou-Yang *et al*., 2020[25] | Quantitative randomised controlled trial | y | y | y | x | n | n | y |
| Ou-Yang *et al*., 2017[26] | Quantitative randomised controlled trial | y | y | x | x | y | n | y |
| Porto-Ferreira *et al*., 2017[27] | Quantitative randomised controlled trial | y | y | x | x | y | n | y |
| Reich *et al*., 2006[28] | Quantitative randomised controlled trial | y | y | x | x | y | n | y |
| Robbins *et al*., 2015[29] | Quantitative non-randomised | y | y | n | y | y | n | y |
| Sarason *et al*., 1992[30] | Quantitative non-randomised | y | y | x | y | x | y | y |
| Sarason *et al*., 1991[31] | Quantitative randomised controlled trial | y | y | x | x | y | n | y |
| Sengeh *et al*., 1997[32] | Quantitative non-randomised | y | y | x | y | x | x | x |
| Sinclair *et al*., 2009[33] | Quantitative randomised controlled trial | y | y | y | n | n | x | y |
| Srzentic *et al*., 2015[34] | Quantitative non-randomised | y | y | y | y | y | n | y |
| Swinyard and Ray, 1977[35] | Quantitative randomised controlled trial | y | y | x | x | y | x | y |
| Ugwu *et al*., 2020[36] | Quantitative non-randomised | y | y | y | y | y | n | y |

Table S3. Risk of bias assessment. Quality-of-studies assessed using the MMAT.

Key: y = yes, n = no, x = inconclusive in the study.

| **Method** | **Outcome measured** | **Result** |
| --- | --- | --- |
| Phone calls | Donation attempt | Mostly significantly improved |
|  | Actual donation rate | Did not improve |
|  | Intention to donate | Improved |
| Educational sessions | Rate of application to become donors | significant improvement |
| Conversational interventions | Odds of donors returning | Increase |
| Web-based | Re-donation rate | Significantly increased |

Table S4. Retaining donors summary of results

| **Authors** | **Date** | **Country and setting** | **Study design** | **Population description** | **Intervention(s)** | **Outcome measured** | **Main findings** | **Funding** | **Declarations** |
| --- | --- | --- | --- | --- | --- | --- | --- | --- | --- |
| Agrawal and Tiwari[1] | 2014 | India  Phone calls | Cross-sectional study | Previous blood donors registered in data base. n=2091 | Retention of blood donors using phone calls. Initial 4 months with no telephone recruiters compared to 2 months with 2 telephone recruiters and 6 months with 3 telephone recruiters. | In-house repeat blood donation numbers. | 60-96% of phone calls were successful (i.e. were answered and expressed interest in donating). 63% in-house donations were prompted by tele-recruitment in this period. 10% of those who said they would donate did. Raw in-house donation numbers increased from 280 before tele-recruitment to 348 with 3 recruiters and 440 with 3 recruiters. | None declared | None declared |
| Bachhotiya *et al*.[2] | 2014 | India  Hospital blood donation centre | Cohort study | 1^st^ prof MBBS & BDS students at Index Medical College. 98 males, 111 females. 36.7% males and 8.2% females had donated blood before. Mean age 18.47 years. n=209 | Students given an interactive awareness session | Willingness to donate blood (mostly for the first time) | Before the awareness session, 88 students were willing to donate blood. After, 196 (93.77% students were willing to donate. | None declared | None declared |
| Bharatwaj *et al*.[3] | 2012 | India  In person | Cohort study | Undergraduate medical students. Mean age 20.4 years old. 49 male, 55 female. Only 1 had previously donated. n=104 | Two-hour interactive awareness session to address issues asked about in a questionnaire given previously. | Willingness to donate blood. | Willingness to donate increased from 85% to 97.1%. | None declared | None declared |
| Bruhin *et al*.[4] | 2015 | Switzerland  Phone call | RCT | Donors in Zurich who had donated at least once before the study. n=15,962 | One group received a phone call 2 days before the blood drive, saying that their blood type was in short supply. The control group received no call. Both groups received a reminder text 1 day before the drive. | Probability to donate and donation rate (repeat donations). | Control group had a donation rate of 30.6%. The phone call increased this by 7.8 percentage points. | Non-industry | None declared |
| Charbonneau *et al*.[37] | 2015 | Canada  Various settings | Cohort study | Ethnic groups in Montreal. | Various awareness-raising activities over several years, including targeted presentations, interviews, and information booths | Number of donors. | 170 Black community donors in 2009 increased to 1582 in 2012. | Non-industry | None declared |
| Chauhan *et al.* [6] | 2018 | India  Classroom | Cross-sectional study | Medical students. 43.4% male, 56.5% female. Average age 20.42 years. 22.9% students had previously donated blood. n=235 | Interactive awareness session using PowerPoint | Self-reported willingness to donate blood (donation and re-donation). | Self-reported willingness to voluntarily donate blood increased from 91% to 97.5% | Non-industry | None declared |
| Clee and Henion[7] | 1979 | United States of America  Phone call | Cohort study | Previous donors that have only donated one time. n=520 | 2x2 factorial design = 4 experiment groups. High or low levels of need in phone call, and either need or implication factors. | Agreeing to donate and attempts to donate blood. | Verbal agreements to donate did not change significantly between the 4 groups from the overall average of 79.3%. Actual donation rate was also not significantly different from overall compliance in the 4 groups. | None declared | None declared |
| Eser *et al*.[8] | 2010 | Turkey  In person | Cross-sectional study | Residents living in Kayseri city centre and university students. Mean age of students: 20 years. N=29614 | Brief education about the need for blood donation. | Rate of admission for blood donation (repeat, as subjects’ data was used from previous records?) | After the education, blood donation rate of admission was 66% in students but only 29% in city residents. | None declared | None declared |
| Ferrari *et al*.[9] | 1985 | United States of America  Phone call | RCT | Freshman and sophomore undergraduates. n=68 | Experimental group: phoned 1-2 nights before a blood drive, asking if they can still be expected to turn up and thanking them. Control: did not receive a prompt call. | Attendance at a college blood drive. | 93% of those phones attended the blood drive, compared to 56% of those not called. | None declared | None declared |
| France *et al.* [10] | 2017 | United States of America  Phone calls | RCT | Mostly whole blood donors (90.3%), 9.7% double red cell donors. 62.4% female. n=484 | Received either a motivational interview (discussing motivations, future intentions etc.) phone call or control phone call (thanking them for donating, reminding them of when next eligible etc.). | Number of attempts at whole blood donation within 60 weeks of previous donation (retention). Levels of motivation | Motivational interview group had significantly less amotivation and larger increases in external and internal motivation. | Non-industry | None declared |
| France *et al.* [11] | 2016 | United States of America  Phone call | RCT | Previous group O, whole blood, or automated red blood cell donors. 63% female, mean age 30 years. n=598 | 6 weeks after recent donation, participants were phone called. Either received an interview (discussing motivations, future intentions etc.) or a control call (thanking them for donating, reminding them of their next date of eligibility etc.). | Motivational autonomy | The motivational interview group had significant increases autonomy and intention, as well as larger reduction in donation anxiety. | Non-industry | None declared |
| Gemelli *et al*.[12] | 2022 | Australia  Donation centre/online | RCT | Deferred donors. 56.6% females, average age 41.2 years. Consisted of novice donors (1-5 previous donations) and established donors (6+ previous donations).  n=6039 | 3 conditions. 1) in-centre brochure + email (included guided conversation led by staff upon deferral). 2) Email only (thanking donor for trying to donate). 3) Control – business-as-usual | Attempts to donate up to 3 months post deferral | Group 1 had increased odds of returning at 3 months (odds ratio = 1.16). No significant difference between groups 2 and 3. Novice donors had OR: 1.38 in group 1, whereas established donors had OR: 1.36 compared to controls. | Non-industry | None declared |
| Germain and Godin[13] | 2016 | Canada  Phone call/online | RCT | Repeat allogenic donors who had provided an email address at a previous donation. 51.5% female, 48.5% male. Mean age 41.8 years. n=3454 | Phone call only (control): received normal phone call from telemarketing department (informing of details of next blood drive). Email only: received same information via email. Phone and email: received both modes of communication, email sent before call. | Registration to give blood | Phone and email had the highest percentage of donors register to give blood (18.45%), compared to phone/control (15.73%) and email (13.2%). This difference was only seen in men. Phone and email did not significantly differ. | Non-industry | None declared |
| Godin *et al.* [14] | 2011 | Canada  Phone call | Cohort study | First-time donors from Quebec. n=1604 | Control: not phoned by agency (B+ and AB+ donors). Experimental group: received a phone call a couple of days before their next opportunity to donate blood (informed location, reviews basic information on how to prepare). | Attempts to give blood in the 12-month follow up. | 48.3% experimental participants attempted to donate compared to 38.0% controls. | None declared | None declared |
| Grassineau *et al*. [15] | 2007 | France  In person | Cohort study | Comorian immigrant community living in Marseilles, France | Non-rigid, semi-structured interviews | Volunteering to donate blood | 92 people volunteered to donate blood during this study. | None declared | None declared |
| Hashemi *et al.* [16] | 2017 | Iran  Various settings |  | Iranian first-time blood donors. 10.4% female, 89.6% male. N=1356 | Received 1 of 6 conditions. 1: reminder phone calls 3 months after first donation, with information about donating again. 2: educational letter 3 months after first donation, containing information on the need for donation/transfusion and how it is used. 3: emotional letter 3 months after first donation, telling a true story about a recipient of transfusion. 4: T-shirt offered as a gift after first donation. 5: 1-hour motivational meeting straight after first donation, informing donor of importance of donation. 6: no intervention | Return rate – attempting second donation within 6 months of the first (donor retention) | Return rate of group 1 = 31.5%, group 2 = 33.2%, 3 = 36%, group 4 = 30%, group 5 = 22% and group 6 = 22.1%. The only significant way to increase donation for female donors was the educational letter group (48.6%; p = 0.003). For males, there was significantly increased return rate in the emotional letter group (36.4%; p = 0.005) and phone recall (23.1%; p = 0.04). | Non-industry | None declared |
| Hayes *et al*. [17] | 1984 | United States of America  Phone call | RCT | Active, inactive, and non-donors. n=910 | Foot-in-door: subjects presented with a small request to encourage involvement towards to target activity (donating blood). Door-in-face: subjects presented with a very large request, followed by the key request (blood donation) as a concession after the initial ask was refused. Control: standard phone call asking to donate blood. All conditions followed a script. | Number of blood donations. | Control was better than door-in-face for all donor types. Foot-in-door induced more donations than control. | None declared | None declared |
| Hu *et al*. [18] | 2022 | China  Online | RCT | Previous donors. Average age 30.47 years, 70.4% males. 69.5% donated once before, 25% twice before. n=751 | Control: received SMS within 6 months of last donation, thanking them for donating/reminding when they are next eligible. Web-based intervention: weekly short videos and web-based responses to questions from donors on WeChat, had real-time communication. | Repeat blood donations | Intervention group had re-donation rate of 16.14%, whereas control was 5.16% (significantly lower). | Non-industry | None declared |
| Jason *et al.* [19] | 1984 | United States of America  Phone call/in person | RCT | Undergraduate students. n=300 | Media only (M): posters/bulletin announcements of blood drive. Media + letter (ML): media and personal letter requesting donation. Media + letter + phone call (MLC): media, personal letter, and phone call.  For the second drive, 7 students were asked to recruit 5 friends and 5 strangers each. | Agreeing to donate and actual donations | 28% of MLC participants agreed to donate, but only 1% did. 1% of M donated and 4% ML donated. No significant difference between the three groups. 13/35 approached friends and 6/29 strangers agreed to donate, but only 11 and 4, respectively, did. | None declared | None declared |
| LaTour and Manrai[20] | 1989 | United States of America  Phone call | Cohort study | Residents of a midwestern community. n=800 | Letter: stating the importance of blood donation. Included a brochure. Informational influence. Phone call: asked the potential donor to come to an upcoming blood drive. Normative influence. Both: received letter first, then phone call 2-3 weeks later | Percentage of people who attended the blood drive and percentage of people who donated. | The letter had no statistically significant effect until combined with normative influence. | None declared | None declared |
| Lipsitz *et al.*  Study 1 [21] | 1989 | United States of America  Phone call | RCT | College students who had signed up to attend a blood drive attending one of three universities in the midwestern metropolitan area. n=156 | Control: received a standard call reminding them of their appointment to donate. Experimental: received the same call, but with a subtle request to commit to going (“We’ll count on seeing you then, ok?”). | Attendance to the blood drive. | The call asking for commitment significantly increased attendance at the blood drive. 81.3% of the experimental condition showed up to donate, compared to 61.8% in the control. | None declared | None declared |
| Lipsitz *et al.*  Study 2 [21] | 1989 | United States of America  Phone call | RCT | College students who had signed up to attend a blood drive attending one of three universities in the midwestern metropolitan area. n=156 | Control: received a standard call reminding them of their appointment to donate. Experimental: received the same call, but instead of “We’ll count on seeing you then, ok?”, students heard either:  “Thank you for caring enough to donate.”, “We’ll mark you on the list as coming. Thank you.” or “We’ll mark you on the list as coming, ok?” (latter two said to reinforce external pressure). | Attendance to the blood drive. | 61.5% who hear the control showed up to donate. For the “Thank you for caring” group, 72.1% showed up to donate. 70% of those told “We’ll mark you on the list.” showed up to donate. When this was turned into a question (“We’ll mark… ok?”), 82.4% attempted to donate – significantly different to control. | None declared | None declared |
| Livitz *et al*. [22] | 2017 | Unites States of America  In person | Cohort study | Young adults, donors, and non-donors from Ohio University undergraduate research pool. 69.4% female, mean age 19.2 years, 90.9% Caucasian. 47.9% had donated at least once before. n=219 | Motivational interview: scripted, addressed motivations, barriers to donating, personal values. Knowledge interview: asked 14 questions on general and specific blood donation knowledge. | Internal motivation and donation intention. | Motivational interview group were more internally motivated post-interview (34% compared to 4% of knowledge group). Motivational interview also had greater increases in intention to donate (mean ± SD, 0.8 ± 1.0 compared to 0.4 ± 1.0 of knowledge group) | None declared | None declared |
| Marsano *et al.,* 2024[23] | 2024 | Germany  Phone call | RCT | First-time donors (mean age 28.8 years) from Leipzig. n=416. | First-time donors were called within a 2 week window, 9 weeks (if male), or 13 weeks (if female) after first donation surveying them about their experience and offering an appointment. Control: usual care. | Six-month return rate of donors. | Return rate was 10.8% higher in the intervention group (65%) than in the control (54.3%). | None declared | None declared |
| Masser *et al*. [24] | 2016 | Australia  Phone call/remote | RCT | Non-donors with appointments to donate. n=3646 | One of 6 conditions from 3 brochure interventions (none, email, hard copy) x 2 national call centre contacts (none, call). Control: current practice of an appointment and hydration reminder text message | Presentation rate for first time donation | Attendance 8.36% higher in NCC call x electronic brochure condition compared to control – the only significant relative risk. | Non-industry | None declared |
| Ou-Yang *et al*. [25] | 2020 | China  Phone call/remote | RCT | Inactive former blood donors. n=11880 | SMS: received a text saying thank you for donating, asking them to donate again. Telephone: scripted interview following a questionnaire. Control: no intervention. | Donation activity for each subject for next 365 days. Occurrence of first next blood donation attempt (reactivation = made at least one donation) | No significant difference in re-donation between the three groups (intention to treat analysis) – 8.1% in telephone group, 8.5% in SMS and 7.4% in control. Average treatment of treated analysis: telephone calls significantly increased re-donation compared to the other groups. For compliers, the re-donation rate for telephone was 11.7% and 8.6% for SMS | Non-industry | None declared |
| Ou-Yang *et al.* [26] | 2017 | China  Phone call/remote | RCT | Inactive donors n=1188 | Phone call: asked why they had stopped donating and whether they would be willing to donate again, following a questionnaire. SMS: received a text saying thank you for donating, asking them to donate again. Control: no intervention | Donation activity in the 7-month follow up period | Re-donation in the three groups was statistically significant, particularly between phone group and control (number of re-donated: 4.8% vs 1.8%, respectively. Rate of re-donation: 7.3% vs 1.8%, respectively). | Non-industry | None declared |
| Porto-Ferreira *et al.* [27] | 2017 | Brazil  Phone call/remote | RCT | First-time donors with serologically reactive screening tests who hadn’t responded to the standard letter. N=484 | Text, new letter, or phone call sent 15 days after standard letter. Script was the same for each intervention (asking donor to return, stating when and to not eat before). | Return rate over 30 days | Phone call had better return rate than text (39.8% vs 28.4%), but phone call was not better than letter (39.8% vs 34.4%). | Non-industry | None declared |
| Reich *et al.* [28] | 2006 | United States of America  Phone call/online | RCT | First-time whole blood donors from two donation centres (Blood Centres of the Pacific (BCP) and United Blood Services (UBS)) n=6919 | 2 (script A or B) x 2 (T-shirt incentive or no incentive) interventions. Received message by email or telephone. Script A: focused on donor empathy, included a story of a patient who needed blood transfusion. Script B: focused on donor self-esteem, mentioning “special people like you”. Both scripts asked to donate again. | Second donations within six months | 20.5% total returned within 6 months. T-shirt was not effective compared to no incentive (20.5% vs 20.6%). Script A was significantly more effective than B (22.2% vs 18.9%). Email significantly less effective than telephone (13.2% vs 27.8%). | Non-industry | None declared |
| Robbins *et al.* [29] | 2015 | United States of America  Online | Cohort study | Black adults (mean age 39.7 years). From 8 states in northeast region. 54% female. 76.7% had donated before. n=150 | Access to programme website for 20-25 minutes. Included pre-test questions, personalised computer-tailored feedback, and post-test questions. | Attitude to donation | Participants were more likely to consider donating after the intervention. | Non-industry | None declared |
| Sarason *et al.* [30] | 1992 | United States of America  Individual class, group of classes or large group setting. In school | Cohort study | High school students. 15% of eligible students had previously donated. N=4970 | Experimental: presented with an 11-minute promotional videotape. In the last 10 minutes of session, filled out a questionnaire. Control: standard presentation with questionnaire at end. | Blood donation rate. Attitude to blood donation. | Videotape associated with 18.7% relative increase in donations. Odds ratio for actual donation (video: control) was 1.58 in those who had never donated before. Video significantly improved attitude to donation. Donation rate for eligible donors who viewed video was 19.7%, vs 16.6% for control | None declared | None declared |
| Sarason *et al*. [31] | 1991 | United States of America  In school | RCT | High school students. n=9378 | Control: normal presentation. 3 experimental presentations, all with a brief introduction, slideshow, then question and answer. Psychological: slides showed donation at high school blood drives, lacked specific informational content. Educational: explained entire donation process, focusing on one donor using photos and diagrams. Combined: used scenes of high school donations and informational content. | Blood donation, attempt to donate. | Psychological intervention (modelling social norms) increased actual donation rate more than the educational or control. The combination and psychological conditions were better at increasing blood donation than educational and control. | None declared | None declared |
| Sengeh *et al.* [32] | 1997 | Sierra Leone  In person | Cohort study | Community members | Community educational campaign, aiming to reduce negative perception of donation/transfusion | Blood donation | Overall increase in number of annual blood donation units compared to pre-intervention | None declared | None declared |
| Sinclair *et al.* [33] | 2009 | United States of America  Phone call | RCT | 127 female, 88 male. Mean age 31.1 years old. Mean of 7 previous donations. N=215 | Adapted motivational interview (AMI): phone call with brief motivational interview following script (discussing individual motivations, concerns etc about donating), then discussed study questionnaires. Control: a brief phone call to discuss questionnaires (no interview) | Intention to donate. Repeat donation attempts at 3-month intervals, tracked for 12 months. | AMI had greater intention to donate as well as higher rate of attempted donation at 12 months (odds ratio 2.48, 95% CI). No significant difference for donation attempts between groups at 3 and 6 months. Slightly higher repeat donation attempts for MAI at 9 months (OR 1.6, 95% CI) | Non-industry | None declared |
| Srzentic *et al.* [34] | 2015 | Serbia  Various settings | Cohort study | University of Belgrade students | Encouraged students to donate using preparatory lectures, discussions, and organised blood drives. Used Facebook profiles, SMS, and media conversation to communicate. | motivation of students to become voluntary blood donors, number of blood donations in the population of Belgrade students and number of blood donations among students. | Increase in average number of students at blood drives from 39 in 2011 to 46 in 2013. There was a 21.3% increase in number of students donating blood in 2013 compared to 2012. | None declared | None declared |
| Swinyard and Ray[35] | 1977 | United States of America  In person/remote | RCT | Female household residents. N=303 | Female household residents contacted personally by a Red Cross volunteer, who delivered a persuasive appeal either with or without a charitable label. Then, received either 0, 1, 2, or 4 mailings with promoting material for the blood centre. Another group received the mailings first, then the personal contact. Control: received mailings but no personal contact | Behavioural/ volunteering intention | Control: intention increased from 5% to ~23%. No advertising and one sales call: 15.4% of group claimed volunteering/ behavioural intention. With more advertising exposure, this increased to 28.6%, 35.9% and 51.5%. Personal contact and one advertising exposure had greater effectiveness than 4 advertising exposures alone. | None declared | None declared |
| Ugwu *et al*.[36] | 2020 | Nigeria  In-person | Cross-sectional study | Undergraduate medical students at Ebonyi State University. 81% within the age 20-25 years. n=158 | Participants given questionnaire to assess attitude to blood donation. Then, an educational workshop intervention. Questionnaire repeated 30 days later. | Attitude to blood donation | The workshop increased proportion of students with positive attitude to donation: 81% were willing to donate blood and 79.1% were willing to encourage their friends/family to donate. | Non-industry | None declared |

Table S5. Summary of included studies, detailing their setting, populations, key methods, results, funding and declarations of interest.

1	Agrawal A, Tiwari A. Tele-recruitment for donor retention. Indian Journal of Hematology and Blood Transfusion. 2014; 30: 22-5.

2	Bachhotiya A, Arora V, Mahashabde P. Evaluation of intervention on voluntary blood donation among 1st prof medical and dental students of index medical college, Indore (MP), India. National Journal of Community Medicine. 2014; 5: 223-6.

3	Bharatwaj R, Vijaya K, Rajaram P. A descriptive study of knowledge, attitude and practice with regard to voluntary blood donation among medical undergraduate students in Pondicherry, India. Journal of clinical and diagnostic research. 2012; 6: 602-4.

4	Bruhin A, Goette L, Roethlisberger A, Markovic A, Buchli R, Frey BM. Call of duty: the effects of phone calls on blood donor motivation. Transfusion. 2015; 55: 2645-52.

5	Charbonneau J, Cloutier M-S, Carrier É. Whole blood and apheresis donors in Quebec, Canada: Demographic differences and motivations to donate. Transfusion and Apheresis Science. 2015; 53: 320-8.

6	Chauhan R, Kumar R, Thakur S. A study to assess the knowledge, attitude, and practices about blood donation among medical students of a medical college in North India. Journal of family medicine and primary care. 2018; 7: 693-7.

7	Clee M, Henion K. Blood donorship and psychological reactance. Transfusion. 1979; 19: 463-6.

8	Eser B, Kurnaz F, Kaynar L, Yay M, Şıvgın S, Ünal A, et al. Are university students a favorable target group for blood donation campaigns? Turkish journal of hematology. 2010; 27.

9	Ferrari JR, Barone RC, Jason LA, Rose T. The effects of a personal phone call prompt on blood donor commitment. Journal of Community Psychology. 1985; 13: 295-8.

10	France CR, France JL, Carlson BW, Himawan LK, Kessler DA, Rebosa M, et al. A motivational interview promotes retention of blood donors with high internal motivation. Transfusion. 2017; 57: 2433-9.

11	France CR, France JL, Carlson BW, Kessler DA, Rebosa M, Shaz BH, et al. A brief motivational interview with action and coping planning components enhances motivational autonomy among volunteer blood donors. Transfusion. 2016; 56: 1636-44.

12	Gemelli CN, Kruse SP, Thijsen A, Van Dyke N, Karki S, Davison TE. Improving donor retention following a temporary deferral: a cluster randomized controlled trial of deferral educational materials. Transfusion. 2022; 62: 1230-9.

13	Germain M, Godin G. The relative efficacy of telephone and email reminders to elicit blood donation. Vox Sanguinis. 2016; 110: 278-81.

14	Godin G, Amireault S, Vézina‐Im LA, Germain M, Delage G. The effects of a phone call prompt on subsequent blood donation among first‐time donors. Transfusion. 2011; 51: 2720-6.

15	Grassineau D, Papa K, Ducourneau A, Duboz P, Boetsch G, Chiaroni J. Improving minority blood donation: anthropologic approach in a migrant community. Transfusion. 2007; 47: 402-9.

16	Hashemi S, Maghsudlu M, Nasizadeh S, Esmaielifar G, Pourfathollah AA. Effective ways to retain first‐time blood donors: a field‐trial study. Transfusion. 2019; 59: 2893-8.

17	Hayes T, Dwyer F, Greenwalt T, Coe N. A comparison of two behavioral influence techniques for improving blood donor recruitment. Transfusion. 1984; 24: 399-403.

18	Hu Q, Hu W, Han W, Pan L. Web-based short video intervention and short message comparison of repeat blood donation behavior based on an extended theory of planned behavior: prospective randomized controlled trial study. Journal of Medical Internet Research. 2022; 24: e37467.

19	Jason LA, Rose T, Ferrari JR, Barone R. Personal versus impersonal methods for recruiting blood donations. The Journal of social psychology. 1984; 123: 139-40.

20	LaTour SA, Manrai AK. Interactive impact of informational and normative influence on donations. Journal of Marketing Research. 1989; 26: 327-35.

21	Lipsitz A, Kallmeyer K, Ferguson M, Abas A. Counting on blood donors: Increasing the impact of reminder calls. Journal of Applied Social Psychology. 1989; 19: 1057-67.

22	Livitz IE, Fox KR, Himawan LK, France CR. A brief motivational interview promotes internal motivation to donate blood among young adults with and without a prior donation history. Transfusion. 2017; 57: 1527-35.

23	Marsano VC, Hasenclever D, Henschler R. The effectiveness of telephone surveys on the return of first‐time donors: A randomized controlled trial. Transfusion. 2024; 64: 1931-9.

24	Masser B, France CR, Foot J, Rozsa A, Hayman J, Waller D, et al. Improving first‐time donor attendance rates through the use of enhanced donor preparation materials. Transfusion. 2016; 56: 1628-35.

25	Ou-Yang J, Bei C-H, Liang H-Q, He B, Chen J-Y, Fu Y-S. Effective methods for reactivating inactive blood donors: a stratified randomised controlled study. BMC Public Health. 2020; 20: 1-11.

26	Ou‐Yang J, He B, Rong X, Bei CH. Can inactive blood donors be re‐recruited? A stratified randomised pilot study. Transfusion medicine. 2017; 27: 421-7.

27	Porto‐Ferreira FA, de Almeida‐Neto C, Murphy EL, Montebello SdC, Nogueira FAH, Koga da Silva EM, et al. A randomized trial to evaluate the use of text messaging, letter, and telephone call reminders to improve return of blood donors with reactive serologic tests. Transfusion. 2017; 57: 102-7.

28	Reich P, Roberts P, Laabs N, Chinn A, McEvoy P, Hirschler N, et al. A randomized trial of blood donor recruitment strategies. Transfusion. 2006; 46: 1090-6.

29	Robbins ML, Paiva AL, Amoyal NR, Brick L, Kessler DA, Burditt C, et al. Acceptability and feasibility of a culturally tailored internet-delivered intervention to promote blood donation in blacks. Health Promotion Practice. 2015; 16: 227-35.

30	Sarason I, Sarason B, Pierce G, Sayers M, Rosenkranz S. Promotion of high school blood donations: testing the efficacy of a videotaped intervention. Transfusion. 1992; 32: 818-23.

31	Sarason IG, Sarason BR, Pierce GR, Shearin EN, Sayers MH. A Social Learning Approach to Increasing Blood Donations 1. Journal of Applied Social Psychology. 1991; 21: 896-918.

32	Sengeh P, Samai O, Sidique S, Kebbie A, Fofana P, Stephens S, et al. Improving blood availability in a district hospital, Bo, Sierra Leone. International Journal of Gynecology & Obstetrics. 1997; 59: S127-S34.

33	Sinclair K, Campbell T, Carey P, Langevin E, Bowser B, Tricaso J, et al. An Adapted Motivational Interview Enhances Donation Attitudes and Increases Retention: SP29. Transfusion. 2009; 49: 63A.

34	Srzentić SJ, Rodić I, Knežević M. The development of the program of voluntary blood donation promotion in students population of the University of Belgrade. Vojnosanitetski pregled. 2015; 72.

35	Swinyard WR, Ray ML. Advertising-selling interactions: an attribution theory experiment. Journal of Marketing Research. 1977; 14: 509-16.

36	Ugwu NI, Uneke CJ, Ugwu CN, Oti WJO, Agbo UN, Akamike IC. Effect of blood donor educational intervention on the knowledge and attitude towards voluntary blood donation among medical students at a Nigerian University. Nigerian Medical Journal. 2020; 61: 163-8.

37	Charbonneau J, Daigneault S. Engaging ethnic minority blood donors. ISBT Science Series. 2016; 11: 140-7.
