# Supplementary material for: The efficacy of interactive communication interventions for motivating blood donation: A systematic review
Source: Vox Sang. 2026 Apr 12;121(8):1110–20. doi: 10.1111/vox.70263 (PMC13453930; doi:10.1111/vox.70263)
Supplement: Supplementary file 2 [file VOX-121-1110-s002.docx]

Appendix

Blood donors, blood donation, blood don*, don* blood, plasma don*, don* plasma, platelet don*, don* platelet*, blood giv*, giv* blood, blood contribut*, blood benefact*, blood drive*, blood banking, Blood Banks, blood bank*, attitude, behavio?r, motivation, drive, intention, motivat*, intention*, intend*, attitude*, view*, perception*, perspective*, opinion*, retention, retain*, health education, health promotion, Education, Teaching, intervention*, program*, campaign*, guidance, incentiv*, stimulus, inducement, educat*, teach*, train*, promot*, instruction*, persuasive communication, communicat*, talk*, conversation*, chat*, discourse, discuss*, persuasion, persuad*, negotiat*, encourag*, interact*, Communication, health communication, Negotiating, verbal behavio?r, speech, Social Interaction, "rais* the topic", "rais* the issue", "rais* the subject", interpersonal relations, professional-patient relations, online social networking, audiovisual aids, remote consultation, telephone, answering services, cell phone, smartphone, text messaging, telemedicine, video*, telephone*, cellphone*, cell* phone*, smartphone*, textmessag*, text messag*, short messag*, sms, direct messag*, messaging, messenger*, telemed*, tele-med*, telehealth, tele-health, ehealth, mobile health, mhealth, m-health, remote consult*, teleconsult*, tele-consult*
